# Qualitative research in the Arabic language. When should translations to English occur? A literature review

**DOI:** 10.1016/j.rcsop.2022.100153

**Published:** 2022-06-24

**Authors:** Nouf M. Aloudah

**Affiliations:** Clinical Pharmacy Department, King Saud University, 11523 PO BOX 50351, Riyadh, Saudi Arabia

**Keywords:** Arabic, Cross-cultural, Qualitative research, Translation, Healthcare, Language, Interviews, Language barriers

## Abstract

Qualitative studies are a valuable approach to exploratory research. Frequently, researchers are required to collect data in languages other than English, which requires a translation process for the results to be communicated to a wider audience. However, language-embedded meaning can be lost in the translation process, and there is no consensus on the optimum timing of translation during the analysis process. Thus, the aim of this paper was to review how researchers conduct qualitative research with Arabic-speaking participants and the timing of data translation.

Three databases were searched (PubMed, Scopus, and Web of Science) for the period January 2010 to January 2020. Studies were excluded if the data collection was not in Arabic or the study was not qualitative or healthcare related.

Thirty-one studies were included, 26 of which translated all transcripts into English and then analyzed the data in English. Five studies transcribed the data in Arabic, analyzed it in Arabic, and then translated the results to English or conducted a parallel analysis. The reason provided for translating the data into English before the analysis was to enable non-Arabic authors to access the data and assist with the analysis. The search results suggest that researchers prefer translating data before analyzing it and are aware of the possibility of losing meaning during the translation process, which might affect the results. A more thoughtful approach to the timing of translation should be undertaken to ensure the subtleties of language are not lost during the analysis of qualitative data.

## Introduction

Qualitative studies are an effective and valuable method for exploratory research as they can identify and provide an in-depth understanding of the issues under study and lead to better intervention designs that may improve healthcare delivery and outcomes.^1^ Qualitative studies attempt to answer many of the ‘why’ queries concerning patients, healthcare providers, and systems that cannot be answered by other research methods.^1^ Qualitative research is meant to gather and analyze non-measurable data about meaning and words.^1^ Thus, it is able to provide information about the ways participants experience the world.^1^

In many instances, researchers must collect data in languages other than English, and using the native language of study participants is preferred.^2^ Collecting data in languages other than English occurs either by conducting research with participants in non-English speaking countries or with foreigners in English-speaking countries.^2^

The Arabic language is widely used when conducting qualitative research in the healthcare field.^3^ Not only is it spoken in 25 countries, but 30% of foreigners in western countries are Arabic-speaking migrants.^4^ However, conducting qualitative research with Arabic participants requires translation for the results to be shared with a wider audience.^3^ Translation from Arabic to English can be conducted either before initiating the analysis (i.e., collecting the data in Arabic and then translating and analyzing it) or after the analysis (i.e., collecting the data in Arabic, analyzing it in Arabic, and then translating the results to English).

Translation involves interpretation of texts that convey the meaning and not solely a word-to-word translation, which overlaps with what the analysis of the qualitative research is about.^5^ Conducting the analysis of the translated data rather than the original data might affect the accuracy of the analysis, as different languages involve different epistemological assumptions and positions.^6^ It is surprising that there is no consensus regarding the optimum timing of translation during qualitative data analysis.^3^^,^^5^, ^6^, ^7^, ^8^

Few attempts have been made to discuss the impact of translation in the context of qualitative research.^5^, ^6^, ^7^^,^^9^ An approach to translate the results rather than the original data was recommended by some researchers.^5^^,^^6^^,^^9^ As qualitative research aims to study meaning, this approach of translating the results and not the original data will assist in being closer to the meaning which participants experienced and shared.^3^^,^^5^^,^^10^

Since the timing and the challenges of translation of qualitative research did not receive the required attention globally and particularly to the Arabic language, the aim of this study was to review how researchers conduct qualitative research with Arabic-speaking participants and the timing of the translations they make.

### Objective

The objective of my study was

1. To map the existing literature of the Arabic-to-English translation for the purpose of sharing the results of a qualitative study with an English-speaking audience
2. To discuss researchers' provided justifications for the to inform practice, research and policymakers.

The research questions were: when does a researcher conduct an Arabic-to-English translation for the purpose of sharing the results of a qualitative study with an English-speaking audience? Do researchers provide a justification for the approach they used?

## Methods

Studies eligible to be included in the review had to use a qualitative study design, be conducted in Arabic, and must have reported the results in English.

### Search method for the identification of studies

#### Electronic searches

**Fig. 1:**
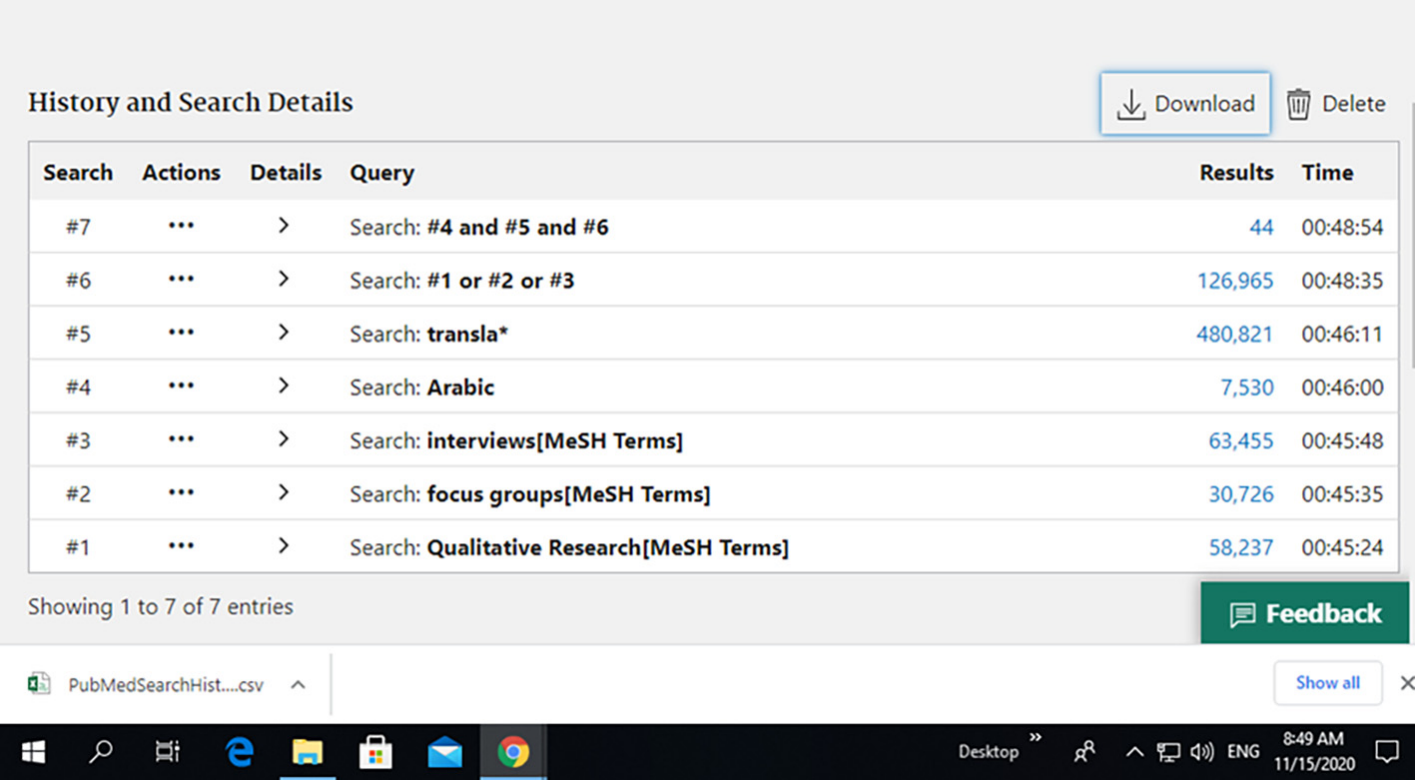
PubMed search results.

Three databases were searched (PubMed, Scopus, and Web of science) for the period January 2010 to January 2020 using the Boolean operators of the following search terms: ‘Arabic or non-English’ and ‘translation’ and ‘qualitative research or interviews or focus groups’. Subject headings for terms describing qualitative research were used. The full search strategy for one of the electronic databases is presented in Table 1 and Fig. 1.

**Table 1:** PubMed Search strategy.

“Arabic”[All Fields] AND “transla*”[All Fields] AND (“qualitative research”[MeSH Terms] OR “interviews as topic”[MeSH Terms] OR “focus groups”[MeSH Terms]) Translations Qualitative Research[MeSH Terms]: “qualitative research”[MeSH Terms] interview[MeSH Terms]: “interviews as topic”[MeSH Terms] focus group[MeSH Terms]: “focus groups”[MeSH Terms]

### Data collection and analysis

#### Study selection

The search and screening were conducted by the author. Abstracts were classified as ‘included’ ‘excluded’ or ‘possible’. Publications that matched the inclusion criteria were retrieved. Full text versions of all relevant and possible versions of studies were examined and decisions were then made for final inclusion.

#### Data extraction

Data were extracted from the full-text studies by the author. Data extraction was conducted using a predesigned Microsoft excel sheet that included the following items: author, year, country, setting, aim, use of theory, data collection method, topic guide development, time of translation, method of analysis, and software used. Further collection of data validity criteria was also applied. The concept of rigor in qualitative research was applied using different criteria^8^^,^^11^. Most criteria are complex and unclear. However, while Lincoln and Guba's (1985) trustworthiness model may not be a gold standard, it is the most explicit and agreed upon in the literature.^11^ Therefore, terms were expanded to include studies if they had mentioned any of the following terms: trustworthiness, reliability, validity, or rigor. The search terms were further expanded to include credibility, transferability, dependability, or confirmability.^8^^,^^11^

**Fig. 2:**
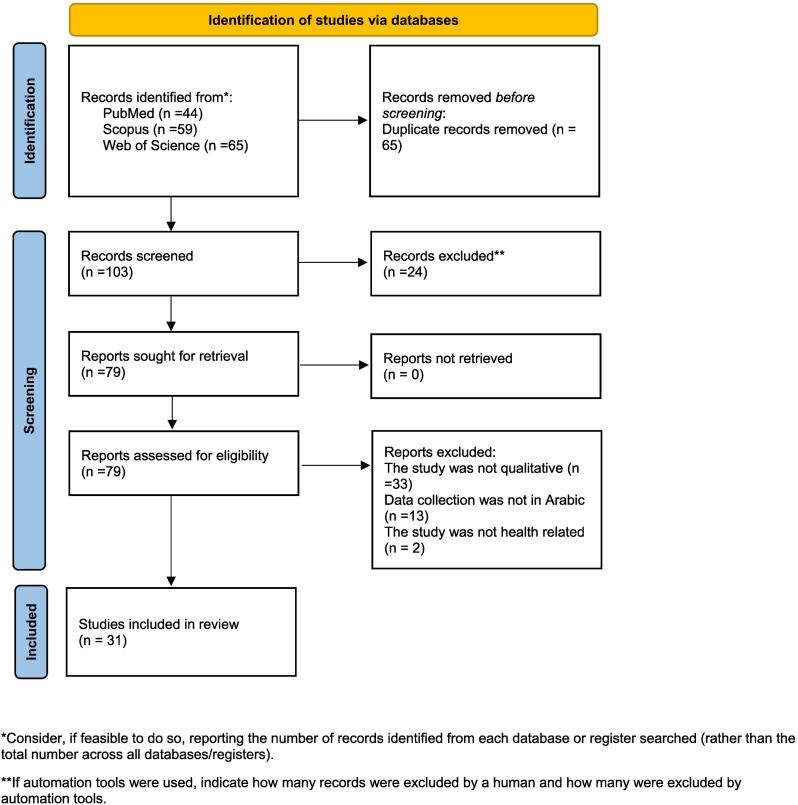
PRISMA 2020 flow diagram. *Consider, if feasible to do so, reporting the number of records identified from each database or register searched (rather than the total number across all databases/registers). **If automation tools were used, indicate how many records were excluded by a human and how many were excluded by automation tools. *From:* Page MJ, McKenzie JE, Bossuyt PM, Boutron I, Hoffmann TC, Mulrow CD, et al. The PRISMA 2020 statement: an updated guideline for reporting systematic reviews. BMJ 2021;372:n71. doi: https://doi.org/10.1136/bmj.n71 For more information, visit: http://www.prisma-statement.org/

Studies were excluded if the data was not in Arabic, not qualitative, or if it was not healthcare-related research. The PRISMA flow chart (Fig. 2) and the PRISMA checklist are presented.

## Results

### Study selection

There were 168 search results. Of those, PubMed contributed 44 results, 59 were from Scopus, and 65 results were from the Web of Science. After removing duplicates, the number of unique articles was reduced to 103. In total, 31 studies were eligible for inclusion. Studies were excluded if the study was not qualitative, it was not health related or the collected data was not in Arabic. Excluded studies and reasons for exclusions are presented in Table 3.

### Study locations

Thirty-one studies were included in the review. The majority were conducted in Australia (n = 11),^12^, ^13^, ^14^, 15., 16, 17, 18, 19, 20, 21, 22 Saudi Arabia (n = 5),^23^, ^24^, ^25^, ^26^, ^27^ Jordan (n = 4),^28^, ^29^, ^30^, ^31^ and 3 studies each were conducted in the USA^32^, ^33^, ^34^ and Qatar.^35^, ^36^, ^37^ Two studies were conducted in Palestine,^38^^,^^39^ and one study each was conducted in Canada,^40^ Sweden^41^ and Turkey.^42^

### Data collection

Empirical data collection methods were either interviews (n = 12) ^13^^,^^18^^,^^20^^,^^21^^,^^23^^,^^25^^,^^26^^,^^28^^,^^32^^,^^34^^,^^39^^,^^41^ or focus groups (n = 14).^12^^,^^14^, 15., 16^,^^20^^,^^22^^,^^27^^,^^29^, ^30^, ^31^^,^^33^^,^^38^^,^^40^^,^^42^ Five studies used both interviews and focus groups,^20^^,^^24^^,^^36^, ^37^, ^38^ and one study used an 18-month long ethnographic study method.^37^ All of the included studies used thematic analysis to evaluate the data.

### Translation process

Twenty-six studies translated all of their transcripts to English and then analyzed them in the English language. The authors were aware of the limitations of this approach as the field work was conducted in Arabic while the analysis used the translated reports.^13^, ^14^, 15., 16^,^^27^^,^^28^^,^^30^, ^31^, ^32^, ^33^, ^34^ The explanation given for using this method was that some of the authors were English-speaking and the translation enabled them to access the data.^13^^,^^14^^,^^30^, ^31^, ^32^, ^33^^,^^39^ Authors mitigated any potential effects of translation on the quality of the analysis by involving bilingual researchers in the coding verification process,^14^ retaining key words in Arabic,^36^^,^^37^ or by having translators add notations to help ensure that the meaning of cultural idioms was not lost.^16^

Authors also clearly mentioned the need to involve professionals when conducting analyses in the language that was spoken and not the translated reports.^13^ Two studies transcribed their data in Arabic, analyzed it in Arabic, and then translated the results to English.^14^^,^^19^^,^^29^ Three studies did parallel analyses^20^^,^^26^^,^^30^; one in Arabic and one in English,^30^ while the other 2 studies conducted some of the analysis in English and the rest in Arabic.^20^^,^^26^ It should be mentioned that these 5 studies involved experts in qualitative analysis who were also Arabic native speakers. Characteristics of the included studies are presented in Table 2.

**Table 2:** Characteristics of included studies.

Author	Year	Country	Aim	Setting	Data collection method	Topic guide	Time of translation	Method of analysis	Software used	Rigor
Albloushi et al	2019	Saudi Arabia	To explore Saudi nursing students' sense of belonging in clinical settings	Fourth year nursing college students	Interviews (n = 16)	NM	Before analysis	Thematic content analysis	Hardcopy, pen, highlighters and Microsoft Word document	Trustworthiness (participants were asked for clarification during interviews. Credibility (the co-authors reviewed some data). Transferability (provided rich, detailed descriptions of the research contexts Dependability (the co-authors reviewed some data to confirm the researcher's findings and the emerging themes and ideas). Conformability (the transcripts and coding were checked and reviewed by the co-authors. Regular follow-up and comparison of codes and themes were carried out. Data were collected over 15 weeks, from three different sites, as noted
Aljuffali 2019	2019	Saudi Arabia	To explore and compare different stakeholder perspectives regarding the safety problems associated with medication supply from community pharmacies in KSA using the HFF	Community pharmacies and their professional and personal network	Interviews (n = 4) and FG (n = 35)	Created using literature and the Human Factor Framework.	Before analysis	Thematic content analysis	Manually and Excel	NM
AlKhaldi et al	2018	Palestine	To identify gaps related to performance and generate insights on how to move forward for health system research performance strengthening	Government institutions, public health universities, health non-governmental organizations	Interviews (n = 52) and 6 FG (n = 104)	Created from literature review	Before analysis	Thematic content analysis	MAXQDA 12 (VERBI GmbH, Berlin)	Trustworthiness (questions were discussed among the research team and piloted, double check of the quality of data analysis and interpretation, all relevant managerial levels and sectors, sample diversity and representation were achieved.
Almansour et al	2020	Saudi Arabia	Exploring the views of health consumers with CVD risk factors regarding their preferences for or willingness to engage with community pharmacy CVD preventive health services.	Community groups	Interviews (n = 25)	Created by the authors		Thematic content analysis	NVivo 11	NM
Alnasser	2015	Saudi Arabia	To explore the proposed features of an Arabic weight loss app by seeking the experiences and opinions of overweight and obese users in order to design a mobile phone app to fit their needs	Community	4 FG (n = 39)	Created by the authors		Thematic content analysis	NM	NM
Aloudah	2018	Saudi Arabia	Explored factors associated with oral hypoglycemic agent's adherence behaviour among patients with Type 2 diabetes	University diabetes clinics	Interviews (n = 20)	created by the author based on Theoretical domain framework (TDM)		Thematic content analysis	Atlas. ti 7 and NVivo 10	NM
Alwan et al	2020	USA	To explore the parental beliefs, perspectives, and practices impacting health and healthcare utilization of Syrian refugee children in a non-traditional migration destination	Refugee resettlement communities	Interviews (n = 18)	Was developed by field experts, local direct providers and other Syrian refugees	Before analysis	Thematic content analysis	Dedoose software	Trustworthiness, credibility, transferability, and confirmability (The emergent themes were reviewed by local community stakeholders, national experts, and the study participants)
Alzayer et al	2017	Australia	To explore the experience and perspectives of Arabic-speaking people with asthma, who have low English proficiency, about their asthma management	Medical practices and community centers	Interviews (n = 25)	Created by the authors from the literature	Before analysis	Thematic content analysis	NM	NM
Alzubaidi et al	2015	Australia	To explore the decision-making processes and associated barriers and enablers that determine access and use of healthcare services in Arabic-speaking and English-speaking Caucasian patients with diabetes in Australia	Healthcare facilities	Interviews (n = 14) and 8 FG (n = 60)	Created by the authors from the literature		Thematic content analysis	NVivo (QSR NUD*IST Vivo: V.8.0)	NM
Amro et al	2019	Qatar	Explored the experiences of clinical research coordinators (CRCs) involved in administering the PANSS using qualitative thematic analysis after focus group discussions.	Hospital	FG	Created by the authors	NM	Thematic content analysis	NM	NM
Arora et al	2018	Australia	To gain an in-depth understanding of Arabic-speaking mothers views on the usefulness of existing oral health education leaflets aimed at young children	Previous study cohort	Interviews (n = 19)	Created by the authors from previous qualitative research	Translation before transcription	Thematic content analysis	NVivo 9 (QSR International Cambridge, MA, USA	Rigor and credibility (five researchers were heavily involved in the data analysis, which included debriefing, transcript coding, and interpretation. Following each interview, the researchers reflected on data collection, summarized the main findings and prepared for subsequent interviews)
Bertrand et al	2015	USA	To understand barriers and facilitators of diabetes self-management education among Arab American patients with diabetes.	Arab Americans community	3 FG (n = 23)	Developed by the authors	Before analysis	Thematic content analysis	NM	NM
Bitar et al	2020	Sweden	To explore Arabic-speaking women's experiences of communication at antenatal care using a Swedish- Arabic App	Antenatal clinics	Interviews (n = 10)	Constructed by the research team with background, open ended and follow up questions	Before analysis	Thematic content analysis	NM	NM
Boughtwood et al	2011	Australia	To examined the experiences and perceptions of the family carers with regard to their caregiving for a person living with dementia	Family carers from culturally and linguistically diverse communities	4 FG (n = 19)	created by the authors	On site translation. Their role was to produce, in English, a transcript of the focus group discussion for data analysis.	Thematic content analysis	Qualitative analysis computer package (not mentioned which one)	NM
Brown et al	2013	Australia	To explore barriers to optimal child restraint use using the integrative behaviour change model in culturally and linguistically diverse communities in New South Wales	Community groups	4 FG	Created by the author using Fishbein's integrative behaviour change model (IBCM)	Before analysis	Thematic content analysis.	NM	NM
Guruge et al	2018	Canada	To explored the health needs of newcomer Syrian women and their experiences in accessing health services	Syrian refugee's agency serving	5 FG (n = 58)	Questions targeting the factors identified in Yang and Hwang's framework	Before analysis	Thematic content analysis	NM	NM
Hunter et al	2018	Australia	to explore cancer survivors' views on integrating traditional and complementary medicine services with conventional cancer care	Hospital clinics	1 FG (n = 11)	created by the authors		thematic content analysis	NVivo Version 11	NM
Huwari et al	2019	Jordan	to explore the major problems faced by Jordanian undergraduate students	University	Interviews (n = 12)	created by the authors	BA	thematic content analysis	NM	NM
Hyatt et al	2018	Australia	To explore low English-speaking patient experiences, preferences, and recommendations regarding a communication package.	Hospital clinics	Interviews (n = 25)	Created by the authors	Before analysis	Thematic content analysis	NVivo 11 (QSR International Melbourne, Victoria, Australia)	NM
Kearns et al	2018	Australia	To explore community perceptions about waterpipe smoking and the health promotion interventions that would be acceptable to Arabic speaking communities	Arabic-speaking community members	10 FG (n = 88)	Created by the authors from literature review	Before analysis	Thematic content analysis	QSR International's NVivo 11	Validity (discussed by the research team and then presented at a meeting with three of the four BCRAs to validate and contextualize the key findings)
Kilshaw et al	2017	Qatar	To gain an in-depth understanding of notions of miscarriage causality and risk among Qataris	Hospital clinics, inpatient rooms, and the early pregnancy unit	18 months of ethnographic study (n = 60)	Created by the authors from previous experience	Before analysis	Thematic content analysis	NM	NM
Malkawi et al	2017	Jordan	To describe the process of developing the Arabic Version of the Preschool Activity Card Sort main aim and described activities unique to their Jordanian children	School children's and their caregivers	6 FG (n = 42)	Created by the authors	after analysis	NM	NM	NM
Marshall et al	2020	Australia	To explore support for infant feeding among Arabic and Chinese speaking migrant mothers in Australia	Health institutions	3 FG (n = 24)	Created based on a refinement framework	Transcribed verbatim in Arabic and Simplified Chinese then translated for analysis	Thematic content analysis	QSR International Pty Ltd., NVivo Qualitative Data Analysis Software, Version 11	Credibility (a sub-set were checked by bi-cultural co-authors)
Nahal et al	2019	Palestine	To explore the lived experience of children with Spina bifida	Rehabilitation centers	Interviews (n = 10)	Created by the authors	Before analysis	Thematic content analysis	NM	Credibility (the different steps described by Lindseth and Norberg (2004) were followed)
Omar	2019	Qatar	The impact of cultural context on the experience of miscarriage.	Women's hospital	18 months ethnographic study (40 interviews and participants observations, n = 60)	Created by the author		Thematic content analysis	NM	Reduced bis (The prolonged involvement and persistent observation of participants deemed to reduce bias)
Saadi et al	2012	USA	To assess the perspectives of Iraqi women refugees on preventive care and perceived barriers to breast cancer screening	Iraqi refugees receiving care at healthcare center	Interviews (n = 20)	Created by authors from previous studies	Before analysis	Thematic content analysis	NM	NM
Saleh et al	2020	Jordan	To explore male Jordanian nurses' experiences of their career in nursing in an Arabic community.	Hospitals	4 FG (each = 5–6 participants)	Created by the authors	Mixed approach	thematic content analysis	NM	Lincolin and Guba criteria
Saleh et al	2012	Australia	To explore the cultural context of cancer, both sporadic and inherited, by examining their beliefs about its causes and the modes of communication about cancer with family, friends and the community	Cancer genetic clinics	Interviews (n = 38)	Created based on the mental distress explanatory model questionnaire	Before analysis	Thematic content analysis	NVivo 8 software package	NM
Scott et al	2014	Australia	To explore knowledge, attitudes and beliefs about lung cancer in three Culturally and linguistically diverse communities in f New South Wales	Cultural and Indigenous Research Centre Australia	2 FG (n = 15)	Created by the author from relevant literature	After analysis	Thematic content analysis	NM	NM
Shoqirat et al	2013	Jordan	tp explore the nurse student's experience of the final year placement and uncovered contributing factors to a positive clinical experience in Jordan	Public university of nursing	2 FG (n = 12)	NM	Before analysis	Thematic content analysis	NVivo 9,	Trustworthiness and credibility (the participants were given the chance to correct the moderator summaries, and they were given the opportunity to add further information). independability and conformability (the involvement of an independent researcher in the process of translation and data analysis achieved). Conformability (enhanced by the bilingual competencies of the). Transferability (was assured by offering the reader sufficient details about the research and how rich descriptions were developed from the data).
Wringe et al	2019	Turkey	To explore the interplay between the disruptions to social trajectories and risks of violence towards Syrian adolescent girls and young women in the context of their displacement to Izmir, Turkey.	Community centers	7 FG (n = 29)	Started with a vignette then asked for participants' views about the scenario	Before analysis	Thematic content analysis	NM	NM

NM = Not mentioned.

**Table 3:** Excluded studies and reasons.

Citation	Year	Reason to exclusion
Abdul Rahman ZAA. The Use of Cohesive Devices in Descriptive Writing by Omani Student-Teachers. SAGE Open. January 2013. doi:10.1177/2158244013506715	2013	The study was not qualitative
Abu Ali RM, Al Hajeri RM, Khader YS, Shegem NS, Ajlouni KM. Sexual dysfunction in Jordanian diabetic women. Diabetes Care. 2008 Aug;31(8):1580-1. doi: 10.2337/dc08-0081. Epub 2008 May 5. PMID: 18458140; PMCID: PMC2494660.	2008	The study was not qualitative
Jamal Ahmad (2017) Arab American parents’ perceptions of their children’s experience in the USA: a qualitative study, Early Child Development and Care, 187:7, 1228-1238, DOI: 10.1080/03004430.2016.1163690	2017	the study was not health related
Al-Abbas, L.S. and Haider, A.S. (2021), "The representation of homosexuals in Arabic-language news outlets", Equality, Diversity and Inclusion, Vol. 40 No. 3, pp. 309-337. doi:10.1108/EDI-05-2020-0130	2020	the study was not health related
Alhabib S, Feder G, Horwood J. English to Arabic translation of the Composite Abuse Scale (CAS): a multi-method approach. PLoS One. 2013 Sep 25;8(9):e75244. doi: 10.1371/journal.pone.0075244. PMID: 24086478; PMCID: PMC3783446.	2013	The study was not qualitative
Al-Haqan, A., Smith, F., Bader, L., Bates, I. 55744866900;7402856186;57188967236;7006628298; Competency development for pharmacy: Adopting and adapting the Global Competency Framework (2020) Research in Social and Administrative Pharmacy,. Cited 1 time.	2020	Data collection was not in Arabic (in English or facilitated in English or in another language)
Alrajhi, Assim. "Static infographics effects on the receptive knowledge of idiomatic expressions." Indonesian Journal of Applied Linguistics [Online], 10.2 (2020): 315-326. Web. 15 Sep. 2021	2021	The study was not qualitative
Alserhan, B.A. 34771182400; Entrepreneurs and trade names: Evidence from the United Arab Emirates(2010) European Business Review, 22 (2), pp. 232-245	2010	The study was not qualitative
Alturki, R., Gay, V.57209294326;15126782000;The development of an arabic weight-loss app Akser Waznk: Qualitative results(2019) JMIR Formative Research	2019	Data collection was not in Arabic (in English or facilitated in English or in another language)
Arnetz BB, Sudan S, Arnetz JE, Yamin JB, Lumley MA, Beck JS, Stemmer PM, Burghardt P, Counts SE, Jamil H. Dysfunctional neuroplasticity in newly arrived Middle Eastern refugees in the U.S.: Association with environmental exposures and mental health symptoms. PLoS One. 2020 Mar 6;15(3):e0230030. doi: 10.1371/journal.pone.0230030. PMID: 32142533; PMCID: PMC7059916.	2020	The study was not qualitative
Author	year	reason to exclusion
Bachner YG. Preliminary assessment of the psychometric properties of the abridged Arabic version of the Zarit Burden Interview among caregivers of cancer patients. Eur J Oncol Nurs. 2013 Oct;17(5):657-60. doi: 10.1016/j.ejon.2013.06.005. Epub 2013 Jul 15. PMID: 23867141.	2013	The study was not qualitative
Baker JR, Raman S, Kohlhoff J, George A, Kaplun C, Dadich A, Best CT, Arora A, Zwi K, Schmied V, Eapen V. Optimising refugee children's health/wellbeing in preparation for primary and secondary school: a qualitative inquiry. BMC Public Health. 2019 Jun 27;19(1):812. doi: 10.1186/s12889-019-7183-5. PMID: 31242897; PMCID: PMC6595577.	2019	Data collection was not in Arabic (in English or facilitated in English or in another language)
Ben Abdelaziz A, Ben Fadhl M, Harrabi I, Ghannem H. [Survey of surgical and radiological semiology modules and their adaptation to the Arabic cultural environment]. East Mediterr Health J. 2003 May;9(3):431-40. Arabic. PMID: 15751937.	2003	The study was not qualitative
Butow PN, Lobb E, Jefford M, Goldstein D, Eisenbruch M, Girgis A, King M, Sze M, Aldridge L, Schofield P. A bridge between cultures: interpreters' perspectives of consultations with migrant oncology patients. Support Care Cancer. 2012 Feb;20(2):235-44. doi: 10.1007/s00520-010-1046-z. Epub 2010 Nov 26. PMID: 21110046.	2012	Data collection was not in Arabic (in English or facilitated in English or in another language)
Churbaji D, Lindheimer N, Schilz L, Böge K, Abdelmagid S, Rayes D, Hahn E, Bajbouj M, Karnouk C. Entwicklung einer Kultursensiblen Version des Mini-International Neuropsychiatric Interview (MINI) in hocharabischer Sprache [Development of a Culturally Sensitive Version of the Mini-International Neuropsychiatric Interview (MINI) in Standard Arabic]. Fortschr Neurol Psychiatr. 2020 Feb;88(2):95-104. German. doi: 10.1055/a-0984-5960. Epub 2019 Dec 18. PMID: 31853910.	2020	The study was not qualitative
Cross-cultural adaptation and validation of Systemic Lupus Erythematosus Quality of Life questionnaire into Arabic. Aziz MM, Galal MAA, Elzohri MH, El-Nouby F, Leong KP. Lupus. 2018 Apr;27(5):780-787. doi: 10.1177/0961203317747714. Epub 2018 Jan 7.PMID: 29308728	2018	The study was not qualitative
Deborah Dubiner, Inas Deeb & Mila Schwartz (2018) ‘We are creating a reality’: teacher agency in early bilingual education, Language, Culture and Curriculum, 31:3, 255-271, DOI: 10.1080/07908318.2018.1504399	2018	The study was not qualitative
El Osta N, Tubert-Jeannin S, Hennequin M, Bou Abboud Naaman N, El Osta L, Geahchan N. Comparison of the OHIP-14 and GOHAI as measures of oral health among elderly in Lebanon. Health Qual Life Outcomes. 2012 Oct 30;10:131. doi: 10.1186/1477-7525-10-131. PMID: 23110518; PMCID: PMC3495839.	2012	The study was not qualitative
Elzahaf RA, Tashani OA, Johnson MI. Prevalence of chronic pain among Libyan adults in Derna City: a pilot study to assess the reliability, linguistic validity, and feasibility of using an Arabic version of the structured telephone interviews questionnaire on chronic pain. Pain Pract. 2013 Jun;13(5):380-9. doi: 10.1111/j.1533-2500.2012.00594.x. Epub 2012 Sep 17. PMID: 22978448.	2012	The study was not qualitative
Ghuloum S, Bener A, Abou-Saleh MT. Prevalence of mental disorders in adult population attending primary health care setting in Qatari population. J Pak Med Assoc. 2011 Mar;61(3):216-21. PMID: 21465930.	2011	The study was not qualitative
Habbash, M., Troudi, S.56893738100;15849237400;The discourse of global English and its representation in the Saudi context: A postmodernist critical perspective(2015)	2015	The study was not qualitative
Hadziabdic, E., Albin, B. & Hjelm, K. Arabic-speaking migrants’ attitudes, opinions, preferences and past experiences concerning the use of interpreters in healthcare: a postal cross-sectional survey. BMC Res Notes 7, 71 (2014). doi:10.1186/1756-0500-7-71	2014	The study was not qualitative
Hamad EO, AlHadi AN, Tremblay PF, Savundranayagam MY, Kinsella EA, Holmes JD, Lee CJ, Johnson AM. Reconstruction of a Caregiver Burden Scale: Identifying Culturally Sensitive Items in Saudi Arabia. Can J Aging. 2018 Jun;37(2):218-233. doi: 10.1017/S071498081800003X. Epub 2018 Apr 2. PMID: 29606174.	2018	The study was not qualitative
Has Arabic Language Learning Been Successfully Implemented? October 2020International Journal of Instruction 13(4):715-730 DOI: 10.29333/iji.2020.13444a	2020	Data collection was not in Arabic (in English or facilitated in English or in another language)
Hasan, A., Fraser, B.J.55964159200;7005454351;Effectiveness of teaching strategies for engaging adults who experienced childhood difficulties in learning mathematics(2015) Learning Environments Research, 18 (1), 13 p.	2015	The study was not qualitative
Hoopman, R., Terwee, C.B., Muller, M.J., Öry, F.G., Aaronson, N.K.15049645000;6603951337;57207400754;6603041296;16940772000; Methodological challenges in quality of life research among Turkish and Moroccan ethnic minority cancer patients: Translation, recruitment and ethical issues (2009) Ethnicity and Health, 14 (3), pp. 237-253	2009	The study was not qualitative
Hyatt, A., Lipson-Smith, R., Schofield, P., Gough, K., Sze, M., Aldridge, L., Goldstein, D., Jefford, M., Bell, M.L., Butow, P.56096416300;57190186627;7202253567;16303622400;35604861100;35321770300;55757206600;6603829897;7401466875;7006662344;Communication challenges experienced by migrants with cancer: A comparison of migrant and English-speaking Australian-born cancer patients(2017) Health Expectations, 20 (5), pp. 886-895	2017	The study was not qualitative
Ibrahim EM, Al-Saad R, Wishi AL, Khafaga YM, El Hussainy G, Nabhan A, Ezzat AA, Ajarim DS, Bazarbashi S, Radwi A, Al-Amro A. Appraisal of communication skills and patients' satisfaction in cross-language encounters in oncology practice. J Cancer Educ. 2002 Winter;17(4):216-21. doi: 10.1080/08858190209528841. PMID: 12556059.	2002	The study was not qualitative
Jomaa L, Naja F, Cheaib R, Hwalla N. Household food insecurity is associated with a higher burden of obesity and risk of dietary inadequacies among mothers in Beirut, Lebanon. BMC Public Health. 2017 Jun 12;17(1):567. doi: 10.1186/s12889-017-4317-5. PMID: 28606120; PMCID: PMC5469040.	2017	The study was not qualitative
Mahdi, M.O.S., Dawson, P. 57205387922;7401859463;The introduction of information technology in the commercial banking sector of developing countries: Voices from Sudan (2007) Information Technology and People, 20 (2), pp. 184-204. Cited 4 times. https://www.scopus.com/inward/record.uri?eid=2-s2.0-34249797323&doi=10.1108%2f09593840710758077&partnerID=40&md5=bbec7e1ec547cda21a4c7edb9a9aaf41 DOI: 10.1108/09593840710758077	2007	The study was not qualitative
Mahyudin Ritonga, Hafni Bustami, Riki Saputra, Rosniati Hakim, Mursal, Shofwan Karim Elhusen, Yoni Marlius. (2020). Reformulating the Arabic Language Teaching Materials Within the Framework of Generating New Cadres of Tarjih and Tajdid Ulama. International Journal of Advanced Science and Technology, 29(7s), 185 - 190. Retrieved from http://sersc.org/journals/index.php/IJAST/article/view/9426	2020	Data collection was not in Arabic (in English or facilitated in English or in another language)
Mangrio E, Zdravkovic S, Carlson E. Refugee women's experience of the resettlement process: a qualitative study. BMC Womens Health. 2019 Nov 27;19(1):147. doi: 10.1186/s12905-019-0843-x. PMID: 31775733; PMCID: PMC6882316.	2019	Data collection was not in Arabic (in English or facilitated in English or in another language)
Panter-Brick, C., Hadfield, K., Dajani, R., Eggerman, M., Ager, A., Ungar, M.7004163686;55595989800;36901937300;6508207734;55859692900;6604059949;Resilience in Context: A Brief and Culturally Grounded Measure for Syrian Refugee and Jordanian Host-Community Adolescents (2018) Child Development, 89 (5), pp. 1803-1820	2018	The study was not qualitative
Pavlovskaya, M., Bier, J.12242589600;25723047700;Mapping census data for difference: Towards the heterogeneous geographies of Arab American communities of the New York metropolitan area(2012) Geoforum, 43 (3), pp. 483-496.	2012	The study was not qualitative
Pia Zeinoun, Natali Farran, Samia J. Khoury & Hala Darwish (2020) Development, psychometric properties, and pilot norms of the first Arabic indigenous memory test: The Verbal Memory Arabic Test (VMAT), Journal of Clinical and Experimental Neuropsychology, 42:5, 505-515, DOI: 10.1080/13803395.2020.1773408	2020	The study was not qualitative
Popper-Giveon A, Schiff E, Ben-Arye E. I will always be with you: traditional and complementary therapists' perspectives on patient-therapist-doctor communication regarding treatment of Arab patients with cancer in Israel. Patient Educ Couns. 2012 Dec;89(3):381-6. doi: 10.1016/j.pec.2012.03.016. Epub 2012 Apr 23. PMID: 22534661.	2012	Data collection was not in Arabic (in English or facilitated in English or in another language)
Reem Abdelhadi, Luma Hameed, Fatima Khaled & Jim Anderson (2020) Creative interactions with art works: an engaging approach to Arabic language-and-culture learning, Innovation in Language Learning and Teaching, 14:3, 273-289, DOI: 10.1080/17501229.2019.1579219	2020	Data collection was not in Arabic (in English or facilitated in English or in another language)
Rifai E, Janlöv AC, Garmy P. Public health nurses' experiences of using interpreters when meeting with Arabic-speaking first-time mothers. Public Health Nurs. 2018 Nov;35(6):574-580. doi: 10.1111/phn.12539. Epub 2018 Sep 17. PMID: 30225947.	2018	Data collection was not in Arabic (in English or facilitated in English or in another language)
Shalash AS, Elrassas HH, Monzem MM, Salem HH, Abdel Moneim A, Moustafa RR. Restless legs syndrome in Egyptian medical students using a validated Arabic version of the Restless Legs Syndrome Rating Scale. Sleep Med. 2015 Dec;16(12):1528-31. doi: 10.1016/j.sleep.2015.07.032. Epub 2015 Sep 25. PMID: 26611951.	2015	The study was not qualitative
Suleiman, M.N., Karim, M.A.56594517800;36442852000;Cycle of bad governance and corruption: The rise of boko haram in Nigeria(2015) SAGE Open	2015	The study was not qualitative
Sweed HS, Elawam AE, Nabeel AM, Mortagy K. Postmenopausal symptoms among Egyptian geripausal women. East Mediterr Health J. 2012 Mar;18(3):213-20. doi: 10.26719/2012.18.3.213. PMID: 22574473.	2012	The study was not qualitative
Tahir, S.Z.B.57195480233;Multilingual behavior of pesantren IMMIM students in makassar(2015) Asian EFL Journal, 2015 (86), pp. 45-64.	2015	The study was not qualitative
Tahir, S.Z.B.57195480233;Multilingual teaching and learning at Pesantren Schools in Indonesia(2017) Asian EFL Journal, 2017 (98), pp. 74-94	2017	The study was not qualitative
Tawash E, Cowman S. The NURSING-Positive Recruitment Arabic Model (NURS-P.R.A.M.): A mixed methods study. J Adv Nurs. 2018 Nov;74(11):2630-2639. doi: 10.1111/jan.13740. Epub 2018 Aug 1. PMID: 29893428.	2018	The study was not qualitative
Thorstensson Dávila, L. 56372436300;The pivotal and peripheral roles of bilingual classroom assistants at a Swedish elementary school (2018) International Journal of Bilingual Education and Bilingualism	2018	Data collection was not in Arabic (in English or facilitated in English or in another language)
Ushama, T.55439841400;Redical Muslim fringe groups' recourse to Takfīr (accusing Muslims of infidelity) a critique(2016) Hamdard Islamicus, 39 (4), pp. 7-42	2016	The study was not qualitative
Weir KEA, Wilson SJ, Gorman DR. The Syrian Vulnerable Person Resettlement Programme: evaluation of Edinburgh's reception arrangements. J Public Health (Oxf). 2018 Sep 1;40(3):451-460. doi: 10.1093/pubmed/fdx109. PMID: 29121218.	2018	Data collection was not in Arabic (in English or facilitated in English or in another language)
Zehetmair C, Nagy E, Leetz C, Cranz A, Kindermann D, Reddemann L, Nikendei C. Self-Practice of Stabilizing and Guided Imagery Techniques for Traumatized Refugees via Digital Audio Files: Qualitative Study. J Med Internet Res 2020;22(9):e17906 (Germany)	2020	Data collection was not in Arabic (in English or facilitated in English or in another language)

Topic guides were described in all of the included studies except for 2.^23^^,^^31^ Topic guides were created by the authors either from the literature or from previous research. The language of the topic guide was described in only one study as having been developed in English and then verbally translated during the interview.^22^ All of the studies used thematic content analysis. An evaluation of trustworthiness and rigor was undertaken in 12 studies, with some authors explicitly describing each term and how they used them.^15^^,^^21^^,^^22^^,^^31^^,^^32^^,^^37^^,^^38^^,^^41^ Saleh et al^30^ mentioned using Lincoln and Guba's criteria without describing how. Others described some aspects of data validation.^23^^,^^35^^,^^39^ Thirteen studies used qualitative analysis computer packages for their data analysis, while 2 studies used pen and paper, Microsoft word, and excel.

## Discussion

This is a scoping review that sought to better understand qualitative research conducted in the Arabic language and the timing of data translation. Research has shown that meanings are embedded in spoken languages and discrepancies may occur during the translation process.^2^^,^^3^^,^^43^ Translation is a vital step for communicating ideas and the results of investigations with the wider, English-speaking community. There is an overlap between translation and analyzing qualitative data as both involve interpretation of a meaning.^5^, ^6^, ^7^^,^^9^ By understanding that the timing of a translation might affect the interpretation of the results, thoughtful consideration of when data should be translated is needed.^2^^,^^3^^,^^43^

The data from 31 studies were reviewed with the aim of identifying when the translation of qualitative data for Arabic language participants occurred. The results indicated that most of the translation process was conducted at an early stage. Typically, researchers translated row data to English, analyzed it in English, and then shared the results. Currently (and unlike quantitative research), there are no guidelines for translating qualitative research.^3^ Thus, it is likely that ambiguous translations would affect the data integrity and research results.^3^^,^^43^^,^^44^

Studies have demonstrated that interpreters are inconsistent when interpreting the same interview at different times,^43^^,^^44^ with differences such as augmentation, summarizing, and omitting information being identified. Hence, it is unclear why, given the added cost and effort, an analysis would be conducted on a translated report as opposed to the original if the primary researchers speak the language of the interviewees.^43^

This review further found that researchers need to verify information in their topic guides. The language used to develop a topic guide, and its translation process, should be described in a study; however, only one study was identified that described the process. Most of the topic guides were developed from the English-language literature and then used with Arabic speakers. This is a step that requires both translation and the cultural adaption of questions to ensure they are appropriate for the participants and do not have explicit details that could weaken a guide's quality^44^.

Only one of the reviewed studies used an 18-month ethnographic approach to collect data (in addition to interviews and focus groups). All of the others used either interviews and focus groups or both. Furthermore, all of the included studies used thematic content analysis. The flexibility of this type of analysis, along with in-depth interviews, can provide rich and detailed results that justifies their use.^38^ However, there are other methods of analysis that can be applied such as observations, grounded theory, and document study^45^^,^^46^, which could enrich qualitative data gathered from Arabic language speakers.^45^^,^^46^ The overwhelming use of interviews, focus groups, and content thematic analyses may indicate that researchers- with the lack guidelines and/or best practices- model their work from the literature and not thoroughly consider the implications.^47^

Evaluating the rigor and trustworthiness of qualitative research is crucial. While it is understood that results might not be generalizable to other settings and there are no agreed upon criteria, there remains the need to assess reliability and validity and to be explicit about how they are assessed.47., 48, 49. In this review, the Guba and Lincoln criteria (with expanded terms) was used as it is currently the most accepted approach.47., 48, 49. Yet, more than half of the included studies did not mention any reliability or validity terms in their publications. While the authors might have used these concepts, this was not explained in the publications, which could limit a reader's ability to trust the study.47., 48, 49. Despite the limitations of not having an agreed upon criteria, it is essential to highlight the importance of documenting the rigorous steps involved in assessing reliability and validity and communicating these steps to improve the strength of the studies.47., 48, 49.

As my results have shown that most of the researcher translated the original data and conducted the analysis on the translated version instead of the original data. This approach might be justified if the authors were not an Arabic language speaker but if both, the participants and the authors are Arabic speakers, it deserves more attention^5^, ^6^, ^7^^,^^9^.

Additionally, beside impacting the validity of the results translation is costly.^49^^,^^50^ Conducting the analysis of the original data will help to narrow the gap between the meaning as experienced by the participants and the meaning interpreted in the finding. Furthermore, this approach will help reducing research cost.

The act of translating row data before an analysis could have been considered acceptable in the past given the lack of computer-aided, qualitative data analysis software (CAQDAS) compatible with Arabic languages. Currently, many of the languages supported by qualitative data software programs, including Atlas.ti^51^ and MAXQDA^52^, are written from right to left (as Arabic is).

### Strengths and limitations

Conducting qualitative research with non-English speakers is challenging. The challenges associated with translating qualitative data have also been discussed in relation to other languages such as Chinese,^5^^,^^9^ German^7^ and Spanish.^49^ One limitation of this study is that it was not a systematic review and, therefore, might have missed other relevant publications. However, while the included studies provided sufficient data, further research that includes systematic reviews should be considered.

Encouraging researchers to analyze data in the language spoken by participants of a qualitative study is infrequent despite it being the original practice. Furthermore, conducting qualitative studies with foreigners speaking Arabic might require a different approach from those conducted in an Arabic country, which could help to validate translations occurring before the analysis. However, researchers should explain the effect of other researchers (authors who can understand Arabic) and build their decision regarding the timing of a translation on that.

Health-related qualitative studies are increasing in Arabic-speaking countries, and a discussion concerning the timing of data translation needs to occur. In addition, increasing the education and training of researchers in analyzing qualitative data is a fundamental step that could result in more qualified qualitative researchers who are able to analyze data in its original form.

## Conclusion

This review explored the process of translation of qualitative health-related data conducted with Arabic-speaking participants. The results suggest that less than 20% of researchers perform an analysis on the original Arabic data. There is a need to thoughtfully decide when the best time to translate data to preserve its meaning occurs.

## Impact of findings on practice statements

• Researchers prefer translating data before analyzing it and are aware of the possibility of losing meaning during the translation process
• A more thoughtful approach to the timing of translation should be undertaken

## Declaration of interests

The author declare that she has no known competing financial interests or personal relationships that could have appeared to influence the work reported in this paper.
